# Omentum-Wrapped Scaffold with Longitudinally Oriented Micro-Channels Promotes Axonal Regeneration and Motor Functional Recovery in Rats

**DOI:** 10.1371/journal.pone.0029184

**Published:** 2011-12-16

**Authors:** Yong-Guang Zhang, Jing-Hui Huang, Xue-Yu Hu, Qing-Song Sheng, Wei Zhao, Zhuo-Jing Luo

**Affiliations:** 1 Institution of Orthopaedics, Xijing Hospital, Fourth Military Medical University, Xi'an, China; 2 476 Clinical Division, Fuzhou General Hospital, Fuzhou, China; 3 Department of Obstetrics and Gynecology, Xijing Hospital, Fourth Military Medical University, Xi'an, China; 4 Department of Biochemistry and Molecular Biology, Basic Medical Science College, Ningxia Medical University, Yinchuan, China; Université de Technologie de Compiègne, France

## Abstract

**Background:**

Tissue-engineered nerve scaffolds hold great potential in bridging large peripheral nerve defects. However, insufficient vascularization of nerve scaffolds limited neural tissues survival and regeneration, which hampered the successful implantation and clinical application of nerve scaffolds. The omentum possesses a high vascularization capacity and enhances regeneration and maturation of tissues and constructs to which it is applied. However, combined application of nerve scaffolds and omentum on axonal regeneration and functional recovery in the treatment of large peripheral nerve defects has rarely been investigated thus far.

**Methods:**

In the present study, an omentum-wrapped collagen-chitosan scaffold was used to bridge a 15-mm-long sciatic nerve defect in rats. Rats that received nerve autografts or scaffolds alone were served as positive control or negative control, respectively. The axonal regeneration and functional recovery were examined by a combination of walking track analysis, electrophysiological assessment, Fluoro-Gold (FG) retrograde tracing, as well as morphometric analyses to both regenerated nerves and target muscles.

**Findings:**

The results demonstrated that axonal regeneration and functional recovery were in the similar range between the omentum-wrapping group and the autograft group, which were significantly better than those in the scaffold alone group. Further investigation showed that the protein levels of vascular endothelial growth factor (VEGF), brain-derived neurotrophic factor (BDNF) and nerve growth factor (NGF) were significantly higher in the omentum-wrapping group than those in the scaffold alone group in the early weeks after surgery.

**Conclusion:**

These findings indicate that the omentum-wrapped scaffold is capable of enhancing axonal regeneration and functional recovery, which might be served as a potent alternative to nerve autografts. The beneficial effect of omentum-wrapping on nerve regeneration might be related with the proteins produced by omentum.

## Introduction

Nerve autografting, the therapeutic gold standard of bridging large nerve defects [1], has some disadvantages including limited donor grafts availability and postoperative complications of donor sites such as scarring and neuroma formation [2]. Therefore, bridging a large nerve defect without sacrificing a healthy nerve to obtain the nerve autograft has significantly clinical importance. Driven by this consideration, extensive research efforts have been made in the field of neural tissue engineering with an attempt to fabricate nerve scaffolds that can guide nerve regeneration as alternatives to nerve autografts. To date, most of the studies have been performed mainly on optimizing the microstructure of nerve scaffolds, or introducing neurotrophic agents and seeding supportive cells. However, tissue-engineered repair strategies mentioned above frequently result in suboptimal nerve regeneration. It has been recognized that insufficient vascularization of nerve scaffolds is among the main factors which limit the performance of nerve scaffolds in promoting nerve regeneration.

Several attempts on improving vascularization of nerve autografts have shown encouraging outcomes in bridging nerve defects [3]–[5]. Nerve autografts with sufficient blood supply survive better, and show beneficial effect on axonal regeneration and functional recovery over those without. In addition to provide sufficient oxygen and nutrients to maintain viability of axonal growth cones and Schwann cells (SCs), the penetration of blood vessels into nerve autografts may also allow the transport of macrophages which stimulate axonal regeneration by secretion and induction a number of growth factors [6]–[8]. All those findings suggest that vascularization and sufficient blood supply to nerve grafts is a key factor which determines their efficacy in bridging large nerve defects. It is therefore proposed that enrichment the degree of vascularization in nerve scaffolds may be of significant importance in enhancing axonal regeneration and functional recovery.

The omentum, the largest peritoneal fold hanging down from the stomach and covering most of the intestines, is a physiologically dynamic tissue and possesses a high vascularization capacity [9]–[11]. Experimentally and clinically, omentum has been widely used as a vascularizing agent in ischemic extremity coverage [12], cardiothoracic reconstruction [13]–[15], brain and spinal cord revascularization [16], [17], and bone healing [18]. Also, studies have shown that omentum significantly promotes vascularization and maturation of tissue-engineered constructs to which it is applied [19]–[22]. In addition, due to the combined ability of angiogenesis and neurotropism, omentum has been used as a viable option for the treatment of the re-operated median nerve following revision carpal tunnel surgery [23]. Nerve defects that bridged by omentum-wrapped nerve autografts showed earlier revascularization and better axonal regeneration compared to those bridged by nerve autografts alone [24]. However, combined application of omentum and tissue-engineered nerve scaffolds on nerve regeneration has been rarely investigated by far.

In the present study, a collagen-chitosan scaffold with longitudinal oriented micro-channels (L-CCH) was fabricated, and was then used to bridge a 15-mm-long sciatic nerve defect in rats. For supporting formation of blood vessels network and nourishing axonal outgrowth across the nerve scaffold, autologous omentum was harvested and wrapped around the scaffold, including the proximal and distal segments of the recipient nerve. The effect of omentum-wrapped scaffold on axonal regeneration and functional recovery was evaluated by both morphological analysis and functional assessment, and the expressions of vascularization and regeneration related genes were evaluated by Western blotting.

## Materials and Methods

### Fabrication of the L-CCH scaffold and microstructure observation

The L-CCH scaffold was prepared following the procedures described previously [25]. In brief, the collagen-chitosan suspension was obtained by mixing and vortexing type I collagen (2.8 wt%; Sigma, St. Louis, MO) and chitosan (0.7 wt%; Sigma) in a solution of acetic acid (0.05 M, pH 3.2) at 4°C. The suspension was then degassed and injected into a hollow, cylindrical copper mold (50.0 mm in length and 2.0 mm in diameter). The mold was vertically placed in a nitrogen canister with its bottom 20 cm above the liquid nitrogen surface and descended gradually at a uniform speed of 2×10^−5^ m/s. After being completely immersed in liquid nitrogen, the suspension was lyophilized for 24 h. The scaffold was then removed from the mold and cut into cylinders (15.0 mm in length and 2.0 mm in diameter). Subsequently, the scaffolds were cross-linked with a solution of genipin (1 wt%, Challenge Bioproducts, Taichung, Taiwan) for 48 h, rinsed three times with distilled water, dehydrated for 30 min with 95% of ethanol, and air dried for 1 week. Finally, the scaffolds were sterilized with an exposure to 20 kGy ^60^Co radiation before surgery.

The cross-linked L-CCH scaffolds were sectioned in longitudinal and transverse planes and observed with a scanning electron microscope (SEM; S-3400N; HITACHI, Tokyo, Japan) at an accelerating voltage of 5 kV. Before observation, the samples were dehydrated using a series of increasing concentrations of ethanol followed by a brief vacuum drying. The dry scaffolds were sputter-coated with gold at 40 mA and then observed under SEM.

### Animals and surgical procedures

Young and male adult Sprague-Dawley rats, weighing approximately 200–220 g, were obtained from Laboratory Animal Centre of Fourth Military Medical University (FMMU) and were divided into three groups as shown in Table 1. Animal surgeries were conducted under a protocol reviewed and approved ethically by Institutional Ethical Committee of FMMU (approval ID: 2010007).

**Table 1:** Number of rats at each time point.

	Autograft Group	L-CCH Group	L-CCH+OW Group
**2 weeks**			
Expression of vascularization and regeneration related genes	4	4	4
**4 weeks**			
Assessment of axonal regeneration and functional recovery*	6	6	6
Expression of vascularization and regeneration related genes	4	4	4
**8 weeks**			
Assessment of axonal regeneration and functional recovery	6	6	6
Expression of vascularization and regeneration related genes	4	4	4
**12 weeks**			
Assessment of axonal regeneration and functional recover	6	6	6
Expression of vascularization and regeneration related genes	4	4	4
Quantification of microvessel density	4	4	4
**Total number**	38	38	38

*Axonal regeneration and functional recovery assessment containing morphometric analysis of sciatic nerve, Fluoro-Gold retrograde tracing, behavioral analysis, electrophysiological assessment, and histological analysis of target muscle.

**Figure 1:**
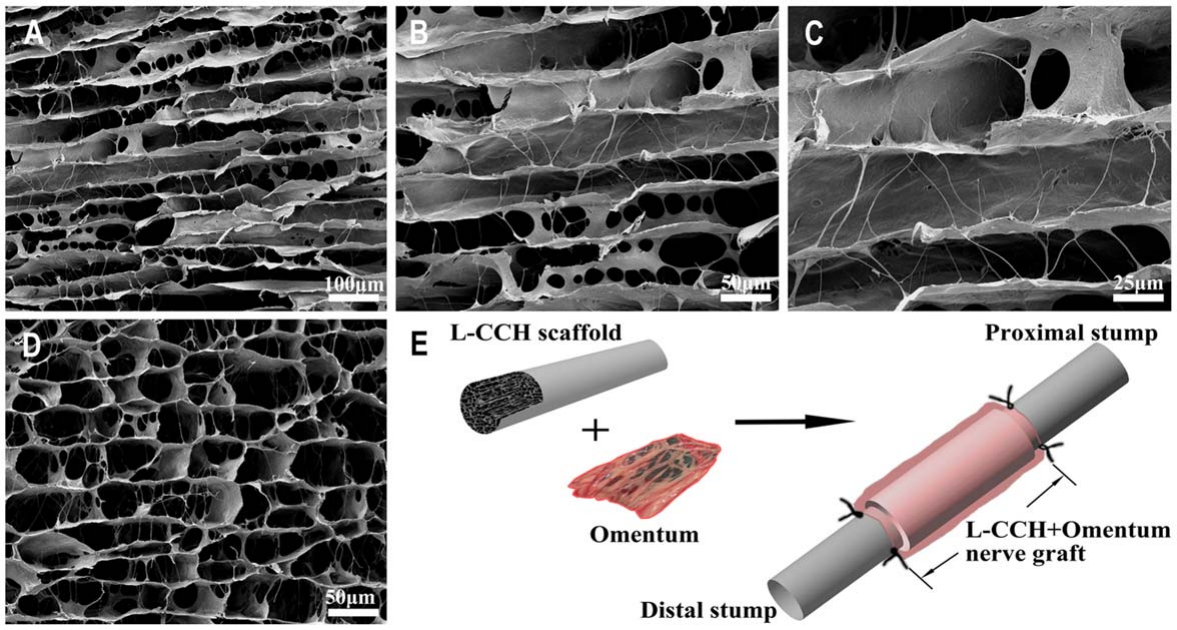
Microstructural appearance of the L-CCH scaffold and schematic diagrams of the omentum-wrapped scaffold. L-CCH scaffold (A–D), Preparation of omentum-wrapped L-CCH scaffold (E).

All animals were anesthetized by an intraperitoneal injection of 1% sodium pentobarbital solution (40 mg/kg body weight). Under aseptic conditions, the left sciatic nerve was exposed using a muscle-splitting incision. A segment of the sciatic nerve was excised, leaving a 15-mm-long defect after retraction of the nerve ends. In the autograft group, the removed nerve segment was rotated 180° and re-implanted with epineural sutures. In the L-CCH group, the nerve defect was bridged with an L-CCH scaffold sutured to both the proximal and distal nerve stumps with 10/0 nylon sutures of monofilament polyamide under 40× magnification. In the L-CCH+omentum-wrapping (OW) group, the nerve defect was first bridged with an L-CCH scaffold as described above. Subsequently, a free omentum flap of the animal was harvested and wrapped completely around the scaffold, including the proximal and distal segments of the recipient nerve (as shown in Figure 1E). The omentum flap was secured to the proximal and the distal nerve sites by 3 perineural 10/0 sutures. In all animals, the skin was closed with 6-0 stitches. After surgery, all animals were returned to their cages, given food and water *ad libitum*.

### Behavioral analysis

Four, 8 and 12 weeks after surgery, walking track analysis was performed on rats and the sciatic functional index (SFI) was calculated as an indicator of functional nerve restoration [26]. In brief, the rats were trained to walk down a wooden track (50×7 cm) into a darkened goal box preoperatively. After surgery, the rats' hind paws were painted with nontoxic finger paint and any changes in their paw prints that resulted from nerve injury and denervation were recorded. The recordings continued until five measurable footprints were collected.

From the footprints, the following parameters were obtained: print length (PL) is the distance from the heel to the top of the third toe, intermediary toe (IT) spread is the distance from the second to the fourth toe, and toe spread (TS) is the distance between the first and the fifth toe. These measures were taken both from the non-operated foot (NPL, NIT and NTS) and from the operated, experimental foot (EPL, EIT and ETS). The SFI was calculated according to the equation: SFI = −38.3×(EPL−NPL)/NPL+109.5×(ETS−NTS)/NTS+13.3×(EIT−NIT)/NIT−8.8. An SFI value that oscillates around 0 indicates better recovery, whereas an SFI value around −100 represents total dysfunction.

### Electrophysiological assessment

Electrophysiological studies were performed after the walking track analysis. The rat sciatic nerve was exposed and the nerve repair site was identified under a surgical microscope after being anaesthetized as mentioned above. The nerve repair area was insulated from the surrounding muscle with a rubber dam and a bipolar stimulating electrode was applied to the nerve trunk at a location 10 mm proximal to the graft site. Compound muscle action potentials (CMAPs) were recorded on the gastrocnemius belly at the ipsilateral side. The peak amplitude of CMAP, latency of CMAP onset, and nerve conduction velocity (NCV) values were calculated.

### Fluoro-Gold (FG) retrograde tracing

After the electrophysiological tests, retrograde labeling was performed and back-labeled cells were counted. In brief, the sciatic nerve was exposed and 5 µl of 4% FG (Biotium, Hayward, CA) solution was intraneurally injected into nerve trunk 5 mm from the distal end of the graft followed by suture of incision. The rats were then kept routinely in their cages for 7 days. Subsequently, the rats were intracardially perfused with 4% (w/v) paraformaldehyde in 0.1 M phosphate buffer under anesthesia as mentioned above. The vertebral canal was opened before the lumbar spinal cord was exposed. The L4, L5 and L6 together with the dorsal root ganglia (DRG) were harvested, postfixed in buffered 4% paraformaldehyde for 4 h, cryoprotected in 30% sucrose overnight at 4°C, and then sectioned on a cryostat. 25 µm thick transverse sections for spinal cords and 16 µm thick longitudinal sections for DRG were mounted on glass slides, viewed and photographed under a fluorescent microscope (BX-60; Olympus). The number of FG-labeled spinal cord motoneurons and the number of FG-labeled DRG sensory neurons were counted directly. Additionally, retrograde labeling was also performed on four non-injured rats and back-labeled cells were counted as normal control monthly.

### Morphometric analysis

After the electrophysiological tests, the regenerated nerves that formed in the place of the grafts were harvested, fixed with 3 wt% glutaraldehyde, and then postfixed in 1% osmium tetroxide in 0.1 M sodium cacodylate buffer (pH 7.3) for 1 h at room temperature. The specimens were dehydrated and embedded in resin, according to standard protocols. Transverse semithin (thickness: 1.0 µm) and ultrathin (thickness: 50.0 nm) sections were prepared from the proximal, middle and distal portions of the regenerated nerve, respectively. The semithin sections were stained with a 1% toluidine blue/1% borax solution prepared in distilled water and examined under a light microscope (AH3; Olympus). Ultrathin sections were stained with uranyl acetate and lead citrate, and examined under a transmission electron microscope (H-600; HITACHI). Morphometric evaluations were completed by examiners who were blind to the experimental design. The parameters measured included (1) the total area of regenerated nerves, (2) the total number of myelinated axons, and (3) the mean diameter of the nerve fibers. The degree of myelination was estimated by the axon to fiber diameter ratio (G-ratio).

### Histological Analysis of Target Muscles

Twelve weeks after surgery, the gastrocnemius muscles were harvested from the operated side of the rats. The muscle specimens were then fixed in buffered 4% paraformaldehyde and subjected to hematoxylin and eosin staining. Thereafter, photographs were taken with a light microscope (AH3; Olympus). For each specimen, the cross-sectional area of muscle fibers was measured by photographs taken from 4 random fields and analyzed with a Leica software package. The extent of the atrophy/reinnervation of target muscles was assessed by the percentage of muscle fiber area (P*_m_*) which was calculated according to the equation: P*_m_* = A*_m_*/A*_t_*×100%, where A*_m_* represents the area of muscle fibers in each field (magnification,×100), and A*_t_* represents the total area including muscle fibers and other tissues such as collagen fibers in the same field as A*_m_*.

### Quantification of microvessel density (MVD)

Twelve weeks after surgery, the regenerated nerves that formed in the place of the grafts were harvested and fixed in buffered 4% paraformaldehyde for 4 h. The specimens were cryoprotected in 30% sucrose overnight at 4°C, and then sectioned on a cryostat. 10 µm thick transverse sections of the middle segment were mounted on glass slides. MVD was quantified by immunofluorescence assay. In brief, specimen-containing slides were immunostained for rabbit anti-CD34 antibody (1∶500, Santa Cruz, USA) at 4°C for 24 h. Next, the primary antibodies were probed with goat anti-rabbit fluorescein isothiocyanate secondary antibody (1∶200; Gibco, Grand Island, NY) for 2 h at room temperature. The specimens were rinsed three times in PBS (pH 7.4) between each step. A fluorescence microscope (BX-60; Olympus, Tokyo, Japan) imaged five random fields from each slide. MVD was counted by an observer blinded to experimental design.

### Expressions of vascularization and regeneration related genes

Two, 4, 8 and 12 weeks after surgery, the regenerated nerves that formed in the place of the grafts were harvested, washed with PBS and lysed with lysis buffer containing protease inhibitors (Promega, Madison, WI, USA). Protein concentration was determined by the BCA protein assay. Protein samples were boiled for 5 min, separated by a 12% SDS-PAGE gel and then transferred onto a PVDF membrane. The membrane was blocked with 5% non-fat dry milk in TBST buffer (50 mM Tris-HCl, 100 mM NaCl, and 0.1% Tween-20, pH 7.4) and incubated with rabbit polyclonal anti-VEGF antibody (1∶500, Santa Cruz, USA), anti-BDNF antibody (1∶1000, Santa Cruz, USA) and anti-β-NGF antibody (1∶800, Santa Cruz, USA) in 5% non-fat dry milk in TBST buffer at 4°C overnight. The membrane was washed with TBST buffer (3×5 min), and incubated with HRP-conjugated goat anti-rabbit IgG (1∶200, Santa Cruz, USA) at room temperature for 2 h. The membrane was then washed with PBS and the HRP activity was determined using an ECL kit (USCNLIFE, USA). The image was visualized by a GS 800 Densitometer Scanner (Bio-Rad, Hercules, CA, USA), and the optical density was determined using PDQuest 7.2.0 software (Bio-Rad, Hercules, CA, USA). Rabbit polyclonal anti-β-actin antibody (1∶500, Santa Cruz, USA) was used as an internal control.

### Data analysis

All data are expressed as the mean±standard error of the mean. The data were analyzed using a factorial ANOVA with the SPSS13.0 software package (SPSS Inc., Chicago, IL). Bonferroni test for pairwise comparisons was used to examine the effect of time and treatments. Differences were considered statistically significant as *P<0.05.

## Results

### Microstructural appearance of the L-CCH scaffold

The microstructure of the L-CCH scaffold resembled the basal lamina micro-channels of normal nerves. The L-CCH scaffold showed longitudinally oriented micro-channels, which were arranged in a honeycomb-like pattern in the cross plane (Figure 1A–D), with a mean cross-sectional diameter of 39.88±9.68 µm (range, 24.32–55.16 µm). In addition, an interconnected porous structure was observed between the micro-channels in the L-CCH scaffold (Figure 1B).

### Behavioral analysis

**Figure 2:**
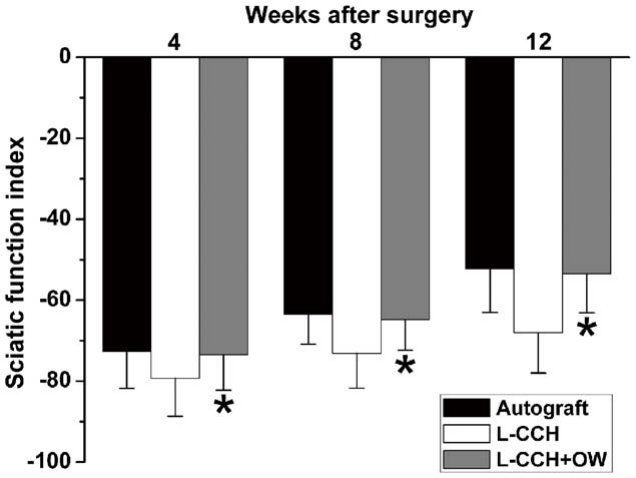
The sciatic function index (SFI) in all groups. All data were expressed as the mean ± standard error of mean. *P<0.05 for the comparison with the L-CCH group.

Rats in all groups showed no signs of regional or systemic inflammation and serious surgical complications after surgery. Good-quality footprints and an increase in SFI measurements were recorded in all groups at 4, 8 and 12 weeks after surgery. The SFI values in the L-CCH+OW group were significantly higher than those in the L-CCH group at 4, 8 and 12 weeks after surgery (*P*<0.05, Figure 2), indicating a better functional recovery achieved in the L-CCH+OW group. In addition, no statistical difference was found in SFI values between the L-CCH+OW and the autograft groups at the predefined time points.

### Electrophysiological assessment

**Figure 3:**
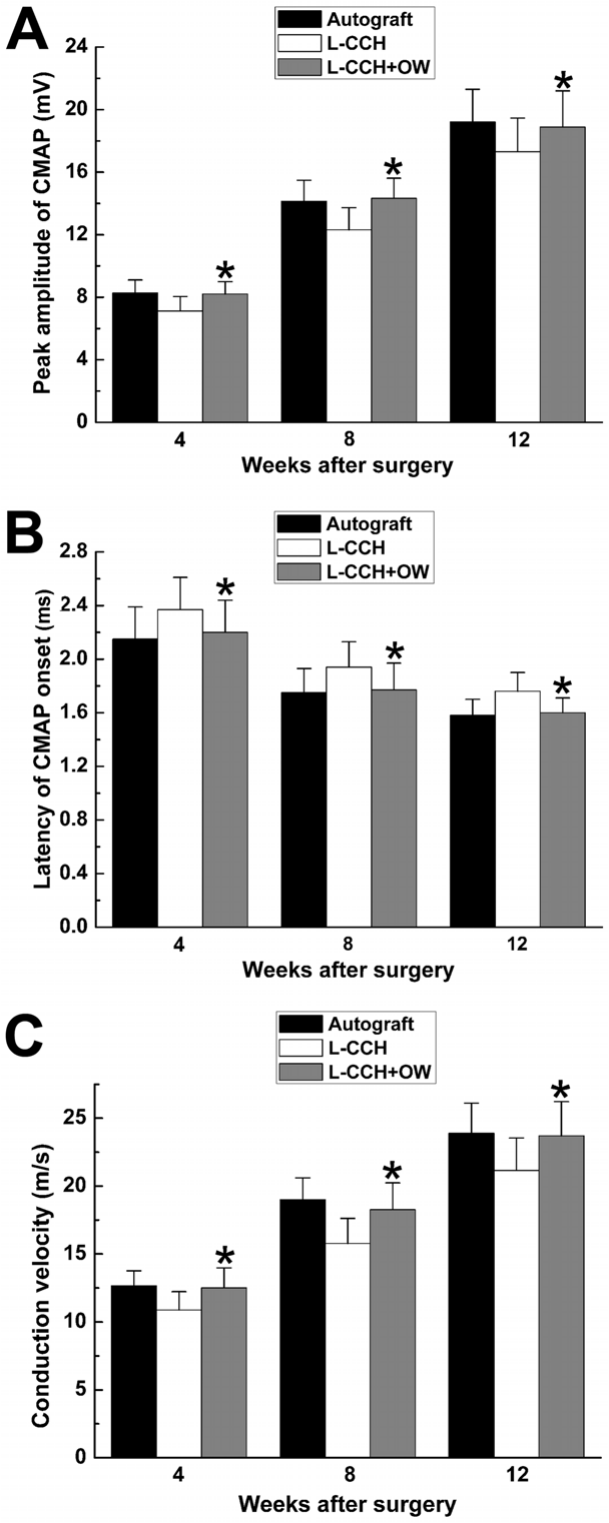
Electrophysiological parameters in all groups. All data were expressed as the mean ± standard error of mean. *P<0.05 for the comparison with the L-CCH group.

Electrophysiological studies showed that motor functional recovery was achieved in all groups at 4, 8 and 12 weeks after surgery. Omentum-wrapping led to a better functional recovery in rats, with a higher peak amplitude of CMAP, higher NCV and shorter latency of CMAP onset in the L-CCH+OW group than those in the L-CCH group (*P*<0.05, Figure 3A–C). The peak amplitude of CMAP, NCV value and latency of CMAP onset were in the similar range between the L-CCH+OW and the autograft groups (*P*>0.05, Figure 3A–C).

### FG retrograde tracing

**Figure 4:**
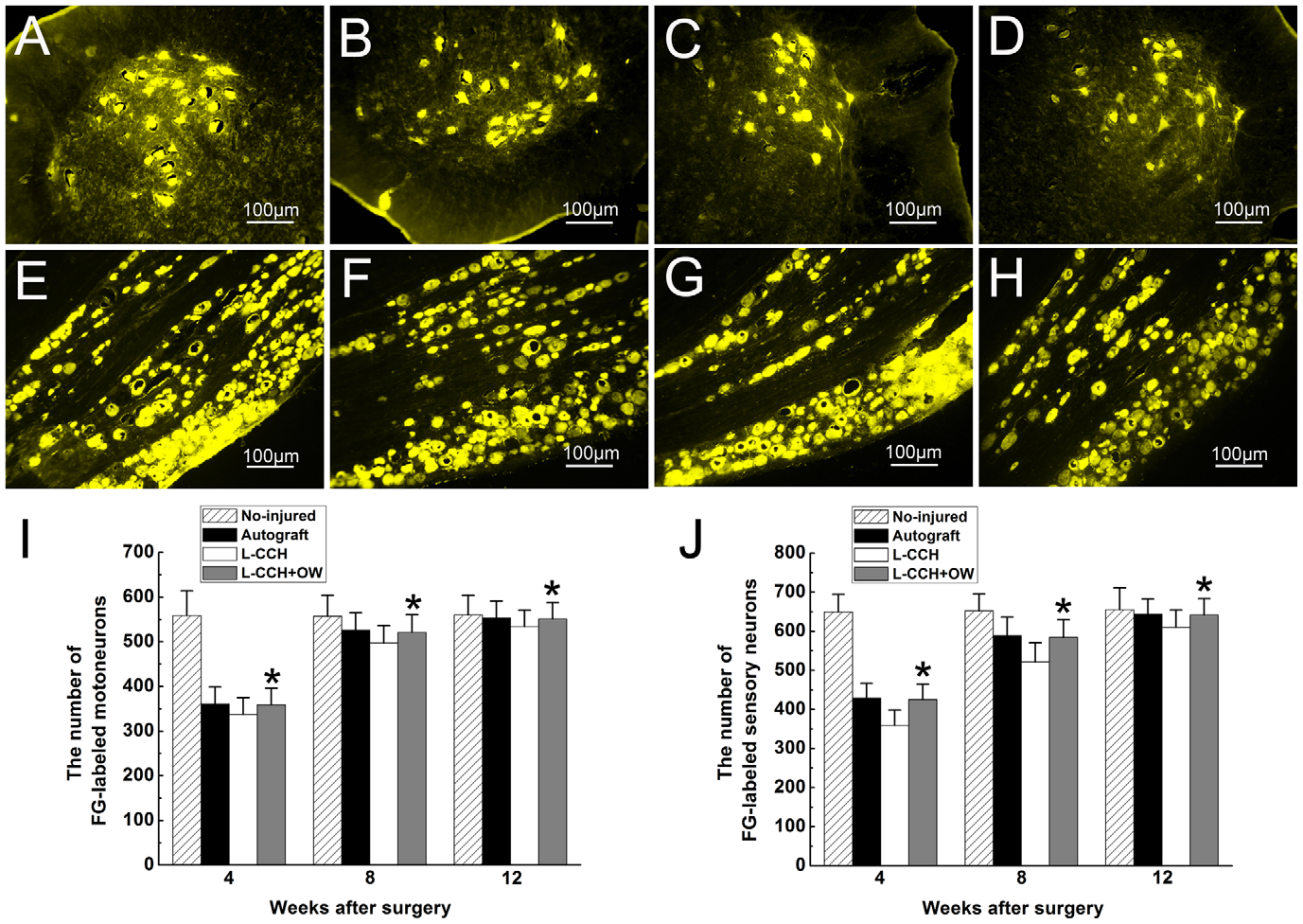
Fluoro-Gold (FG) retrograde tracing. FG-positive motoneurons in spinal cord (A–D) and sensory neurons in DRG (E–H) in the no-injured group (A, E), autograft group (B, F), L-CCH group (C, G), and L-CCH+OW group (D, H) at 12 weeks postoperatively. The total number of FG-positive motoneurons (I) and sensory neurons (J) which regenerated into distal nerve stump was plotted as a function of time postoperatively. All data are expressed as the mean ± standard error of mean. *P<0.05 for the comparison with the L-CCH group.

FG-positive cells were observed within both the anterior horn of spinal cord and DRG in all groups. The number of FG-labeled motoneurons and sensory neurons were not significantly different between the L-CCH+OW and the autograft groups (*P*>0.05, Figure 4B, D, F, H–J), which were significantly higher than those in the L-CCH group (*P*<0.05, Figure 4B–D, F–J) at 4, 8 and 12 weeks after surgery. This finding indicates that more motoneurons and sensory neurons survived and more nerve fibers successfully regenerated into the distal stumps in the L-CCH+OW and the autograft groups compared with those in the L-CCH group.

### Morphometric analysis

**Figure 5:**
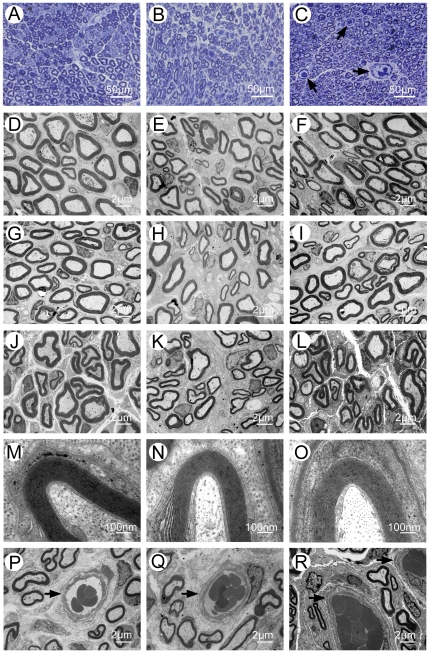
Morphometric analysis of regenerated nerves. Toluidine blue staining of regenerated axons (A–C), myelin sheath (M–O), and blood vessels (P–R) at the distal portion and transmission electron micrographs of regenerated axons (proximal portion: D–F; middle portion: G–I; distal portion: J–L) in the autograft group (A, D, G, J, M, P), L-CCH group (B, E, H, K, N, Q), and L-CCH+OW group (C, F, I, L, O, R) at 12 weeks postoperatively. Blood vessels are showed by arrows in (C and P–R).

**Figure 6:**
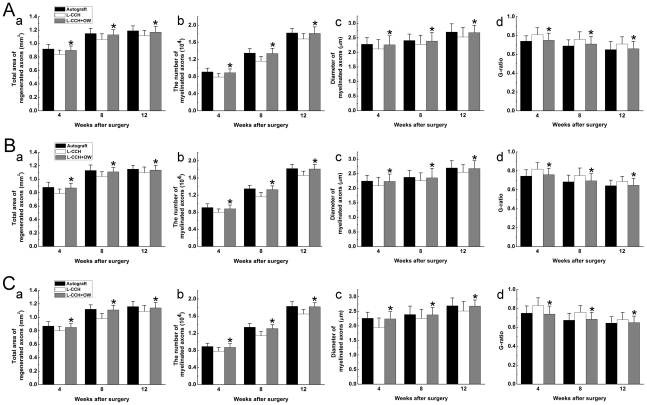
Morphometric evaluations of regenerated nerves. A: the proximal portion; B: the middle portion; C: the distal portion. The cross-sectional area of regenerated nerve [A(a)–C(a)], quantification of the myelinated axons [A(b)–C(b)], the diameter of myelinated axons [A(c)–C(c)], and the G-ratio [A(d)–C(d)]. All data are expressed as the mean ± standard error of mean. *P<0.05 for the comparison with the L-CCH group.

**Figure 7:**
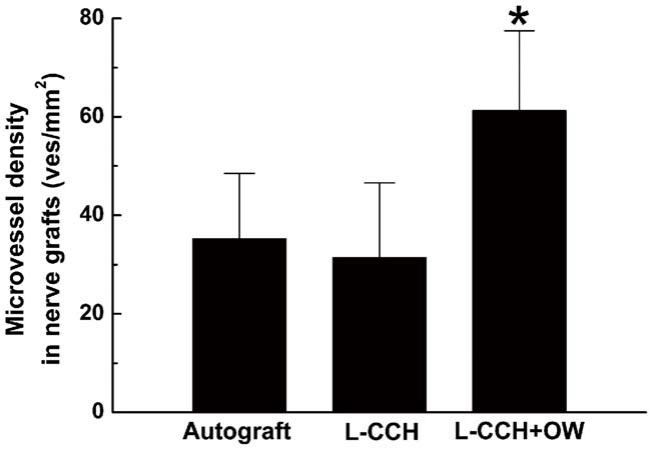
Quantification of microvessel density. All data are expressed as the mean ± standard error of mean. *P<0.05 for the comparison with the L-CCH group.

The omentum-wrapped L-CCH scaffold was capable of enhancing axonal regeneration. Regenerated nerves were observed in each group at 4, 8 and 12 weeks after surgery (Figure 5A–C). To better understand nerve regeneration over the whole length of nerve grafts, transverse images of transmission electron microscopy were taken from the proximal, middle and distal portions of the regenerated nerves, respectively. According to transmission electron microscopy for all groups, regenerative myelinated axons were observed in each portion of nerve grafts with a uniform structure of myelin sheath (Figure 5D–L). In the transverse sections of the L-CCH+OW and the autograft groups, massive myelinated axons were exhibited with an even distribution in each portion, while in the L-CCH group, the regenerated axons showed scattered distribution accompanied by decrease in density in each portion. In the proximal, middle, and distal portions of the regenerated nerves, the total area of regenerated axons [Figure 6A(a)–C(a)], the total number of myelinated axons [Figure 5B, C; Figure 6A(b)–C(b)], the mean diameter of the myelinated axons [Figure 5N, O; Figure 6A(c)–C(c)], and the degree of myelination [G-ratio, Figure 6A(d)–C(d)] were significantly higher in the L-CCH+OW group than those in the L-CCH group (*P*<0.05, Figure 6A–C). In addition, mature blood vessels were also found in all three groups (Figure 5P–R). The MVD in the L-CCH+OW group was significantly higher than that in both the L-CCH and the autograft groups at 12 weeks after surgery (*P*<0.05, Figure 7).

### Histological Analysis of Target Muscles

**Figure 8:**
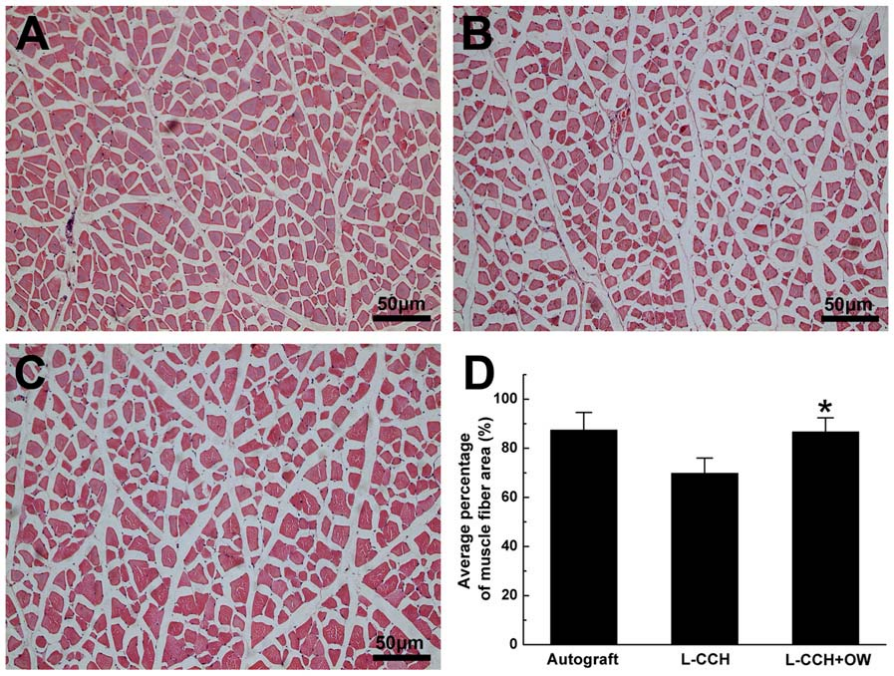
Histological analysis of target muscle. Light micrographs of the transverse-sectioned gastrocnemius muscle following hematoxylin and eosin staining for the operated side of the autograft group (A), L-CCH group (B), and L-CCH+OW group (C) at 12 weeks postoperatively. The average percentages of muscle fiber for each group are shown in (D). All data are expressed as the mean ± standard error of mean. *P<0.05 for the comparison with the L-CCH group.

Morphometric analysis of gastrocnemius muscles showed that the atrophy of muscle in the L-CCH+OW group was to some extent reversed. The average cross-sectional area of muscle fibers were in the similar range between the L-CCH+OW and the autograft groups, which were significantly higher than that in the L-CCH group at 12 weeks after surgery (*P*<0.05, Figure 8).

### Expression of vascularization and regeneration related genes

**Figure 9:**
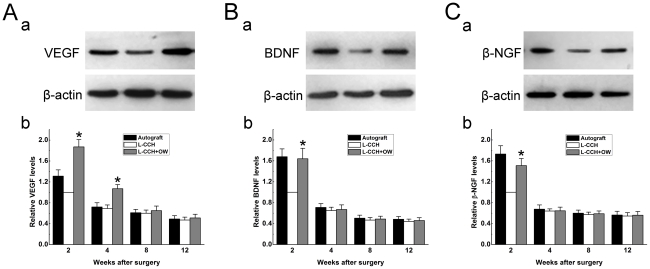
Protein levels of VEGF, BDNF and β-NGF. A(a)–C(a): VEGF, BDNF and β-NGF immunoreactivity on Western blots at 2 weeks postoperatively. A(b)–C(b): relative protein levels of VEGF, BDNF and β-NGF among groups at 2, 4, 8 and 12 weeks postoperatively. Each test was repeated three times. *P<0.05 for the comparison with the L-CCH group.

Omentum-wrapping increased the protein levels of angiogenic and neurotrophic factors within the L-CCH scaffold. As shown in Figure 9, the protein levels of VEGF in the L-CCH+OW group were 1.87 and 1.55 times higher than those in the L-CCH group at 2 and 4 weeks after surgery, respectively [*P*<0.05, Figure 9A(a, b)]. The VEGF levels were in the similar range at 8 and 12 weeks after surgery between the L-CCH+OW group and the L-CCH group. The protein levels of BDNF and β-NGF in the L-CCH+OW group were 1.64 and 1.51times higher than those in the L-CCH group at 2 weeks after surgery, respectively [*P*<0.05, Figure 9B(a, b), C(a, b)]. The protein levels of BDNF and β-NGF in the L-CCH+OW group were not significantly different from those in the L-CCH group at 4, 8 and 12 weeks after surgery.

## Discussion

In the present study, we investigated the effect of the omentum-wrapped L-CCH scaffold on axonal regeneration and functional recovery in bridging a 15-mm-long sciatic nerve defect in rats. Our study showed that omentum-wrapped scaffold significantly enhanced axonal regeneration and functional recovery. In addition, application of omentum was able to support formation of blood vessels network and significantly increased the protein levels of VEGF, BDNF and NGF within the scaffold in the early weeks after surgery. These findings indicate that the omentum-wrapped scaffold is capable of vascularizing nerve scaffold which might be able to nourish axonal outgrowth, hence improving axonal regeneration and motor functional recovery.

Local vascular supply is essential in the regenerative environment of injured peripheral nerves, while neovascularization may further enhance axon-SC association and play an important role during the process of nerve regeneration [27]–[30]. Therefore, vascularization of nerve scaffolds is crucial for axonal outgrowth and restoration of functional recovery. In the present study, the omentum-wrapping significantly enhanced axonal regeneration through the whole length of the L-CCH scaffold. Massive myelinated axons were exhibited with an even distribution in the proximal, middle, and distal portions of nerve grafts in the L-CCH+OW group at 4, 8 and 12 weeks after surgery. In each portion of the nerve grafts, the number of myelinated axons in the L-CCH+OW group was significantly higher than that in the L-CCH group at the predefined time points after surgery. In addition, the number of FG-labeled motoneurons and sensory neurons in the L-CCH+OW group was as well significantly higher than that in the L-CCH group. These mean that the “axon-SC dance” [31] may be supported to some extent by application of omentum, and more neurons may be supported survival and more neurites may be generated by regenerating neurons.

The introduction of omentum-wrapping led to improved motor functional recovery. The amplitude of CMAP as well as the histological appearance of target muscles reflects the reinnervation of distal target muscles. In the present study, both the amplitude of CMAP and the histological appearance of gastrocnemius muscles in the L-CCH+OW group were significantly higher than those in the L-CCH group. This indicates that more axons may successfully proceed through the omentum-wrapped scaffold into the distal stumps and reinnervate target muscles, therefore the atrophy of target muscles was partially reversed. In addition, walking track analysis was performed and scored by SFI which provides a reliable measure for evaluating the recovery of motor function. In the present study, the SFI values were in the similar range between the L-CCH+OW and the autograft groups. Nerve autograft is the most frequently used positive control in studies of nerve defects reconstruction. In many previous studies, incorporating neurotrophic factors or introducing supportive cells into nerve scaffolds can achieve a similar performance to nerve autograft in promoting nerve regeneration and functional recovery. Therefore, it is reasonable to speculate that the efficacy of the omentum-wrapped L-CCH scaffold in promoting nerve regeneration might be further improved by introducing supportive cells and incorporating neurotrophic factors into the scaffold.

It is reported that an omentum on contact with a foreign body or activated by injury expands rapidly in size and mass, and the tissue growth is paralleled by the increase in blood vessel density, thereby supporting increased angiogenesis [10]. Vascular endothelial growth factor (VEGF) is the primary angiogenic factor produced by omentum, which may facilitate the growth of new blood vessels and accelerate tissue repair [10], [19]–[21], [32], [33]. In the present study, a significantly higher blood vessel density was observed in the L-CCH+OW group compared with that in the L-CCH group. Further investigation found that the protein levels of VEGF in the L-CCH+OW group were significantly higher than those in the L-CCH group at 2 and 4 weeks after surgery, which might be responsible for the formation of higher blood vessel density, thereafter providing adequate blood supply to the regenerating axons and consequently resulting in improved nerve regeneration and functional recovery. In addition, VEGF has been reported to have neurotrophic and mitogenic activity on growth cones and SCs [34]–[36], hence stimulating axonal outgrowth, survival and proliferation of SCs independent of the increased vascularization. Therefore, the angiogenic activity and neurotrophic property of VEGF might contribute to the beneficial effect of omentum on axonal regeneration and functional recovery.

Brain-derived neurotrophic factor (BDNF) and nerve growth factor (NGF) hold great potential in promoting nerve regeneration by providing an appropriate environment for axonal outgrowth [37]. Many studies have shown that up-regulation of neurotrophic factors during nerve repair process is beneficial for nerve regeneration [38], [39]. In the present study, both the protein levels of BDNF and NGF were significantly higher in the L-CCH+OW group than those in the L-CCH group at 2 weeks after surgery, which might be, at least in part, responsible for the improved nerve regeneration in the L-CCH+OW group. Despite the finding that elevated protein levels of BDNF and NGF was observed in the L-CCH+OW group, the source of BDNF and NGF was not identified in the present study. SCs regain the ability of synthesizing neurotrophic factors after peripheral nerve injury, thus are probably the main source of BDNF and NGF. In addition, omentum might be served as a pool for BDNF. It has been shown that BDNF expression was noted in vascular endothelial cells, which were abundantly found in omentum [40], [41]. The omentum implanted at the local site of nerve scaffold might contribute to the up-regulation of BDNF observed in the L-CCH+OW group. The mechanism underlying the up-regulated expression of BDNF and NGF needs to be clarified in the future studies.

Large peripheral nerve defects are frequently caused by trauma, and patients with those injuries should be advised to seek emergency surgery immediately. Otherwise, the dispersed axonal growth would lead to neuroma formation, and the atrophy of denervated target organs would increase the risk of permanent disability. Autologous omentum is not only free of ethical issues but also easily harvested through laparoscopic techniques without many intraabdominal complications associated with laparotomy [15]. In addition, omentum is easy to integrate with local sites and avoid questions regarding immunogenicity, thus exhibits therapeutic potential in the immediate repair of large nerve defects while combined with nerve scaffolds. The encouraging outcomes in the present study indicate that the combined usage of omentum and nerve scaffolds, if further confirmed in larger animals and even humans, may serve as a potent alternative to nerve autografts. Moreover, nerve autograft contains Schwann cells and basal lamina micro-channels, which are responsible for axonal regeneration achieved by nerve autograft [42], while the longitudinally oriented micro-channels within the L-CCH scaffold and omentum wrapped around the channels might largely account for axonal regeneration achieved by the omentum-wrapped L-CCH scaffold. Although the nerve regeneration achieved by omentum-wrapped L-CCH scaffold is not superior to that by nerve autograft, it is still encouraging that these two grafts achieved similar performance in promoting nerve regeneration in the present study. It can be hypothesized that seeding SCs and incorporating neurotrophic factors into the omentum-wrapped L-CCH scaffold may achieve better nerve regeneration and functional recovery than nerve autograft, which will be investigated in our future studies.

In conclusion, the combined usage of omentum and the L-CCH scaffold described here has several potential advantages over other strategies for promoting large nerve defect regeneration. Firstly, the L-CCH scaffold is relatively easy to prepare, handle, store, and sterilize. Secondly, the longitudinally oriented micro-channels in the L-CCH scaffold are capable of guiding the linear growth of regenerated axons, and the interconnected porous structure may facilitate penetration of blood vessels. Thirdly, omentum potentiates vascularization capacity on nerve scaffolds and neurotrophic effect on axonal regeneration, which could be applied in emergency surgery of large peripheral nerve defects. Further studies attempt to combine omentum-wrapping with several other strategies, such as optimizing the microstructure of the L-CCH scaffold, incorporating neurotrophic agents, and seeding supportive cells.
